# Genomic network analysis of environmental and livestock F-type plasmid populations

**DOI:** 10.1038/s41396-021-00926-w

**Published:** 2021-03-01

**Authors:** William Matlock, Kevin K. Chau, Manal AbuOun, Emma Stubberfield, Leanne Barker, James Kavanagh, Hayleah Pickford, Daniel Gilson, Richard P. Smith, H. Soon Gweon, Sarah J. Hoosdally, Jeremy Swann, Robert Sebra, Mark J. Bailey, Timothy E. A. Peto, Derrick W. Crook, Muna F. Anjum, Daniel S. Read, A. Sarah Walker, Nicole Stoesser, Liam P. Shaw, REHAB consortium, Manal AbuOun, Muna F. Anjum, Mark J. Bailey, H. Brett, Mike J. Bowes, Kevin K. Chau, Derrick W. Crook, Nicola de Maio, Nicholas Duggett, Daniel J. Wilson, Daniel Gilson, H. Soon Gweon, Alasdair Hubbard, Sarah J. Hoosdally, William Matlock, James Kavanagh, Hannah Jones, Timothy E. A. Peto, Daniel S. Read, Robert Sebra, Liam P. Shaw, Anna E. Sheppard, Richard P. Smith, Emma Stubberfield, Nicole Stoesser, Jeremy Swann, A. Sarah Walker, Neil Woodford

**Affiliations:** 1 Nuffield Department of Medicine, University of Oxford, Oxford, UK; 2 Animal and Plant Health Agency, Weybridge, Addlestone UK; 3 UK Centre for Ecology & Hydrology, Wallingford, UK; 4 University of Reading, Reading, UK; 5 Icahn Institute of Data Science and Genomic Technology, Mt Sinai, NY USA; 6 NIHR HPRU in Healthcare-Associated Infection and Antimicrobial Resistance, University of Oxford, Oxford, UK; 7 NIHR Oxford Biomedical Research Centre, University of Oxford, Oxford, UK; 8 Animal and Plant Health Agency, Weybridge, Addlestone UK; 9 UK Centre for Ecology & Hydrology, Wallingford, UK; 10 Thames Water Utilities, Clearwater Court, Vastern Road, Reading, UK; 11 Nuffield Department of Medicine, University of Oxford, Oxford, UK; 12 NIHR HPRU in Healthcare-Associated Infection and Antimicrobial Resistance, University of Oxford, Oxford, UK; 13 NIHR Oxford Biomedical Research Centre, University of Oxford, Oxford, UK; 14 Wellcome Trust Centre for Human Genetics, University of Oxford, Roosevelt Drive, Oxford, UK; 15 University of Reading, Reading, UK; 16 Department of Tropical Disease Biology, Liverpool School of Tropical Medicine, Liverpool, UK; 17 Icahn Institute of Data Science and Genomic Technology, Mt Sinai, NY USA; 18 Antimicrobial Resistance and Healthcare Associated Infections (AMRHAI) Reference Unit, National Infection Service, Public Health England, London, UK

**Keywords:** Environmental microbiology, Genomics

## Abstract

F-type plasmids are diverse and of great clinical significance, often carrying genes conferring antimicrobial resistance (AMR) such as extended-spectrum β-lactamases, particularly in *Enterobacterales*. Organising this plasmid diversity is challenging, and current knowledge is largely based on plasmids from clinical settings. Here, we present a network community analysis of a large survey of F-type plasmids from environmental (influent, effluent and upstream/downstream waterways surrounding wastewater treatment works) and livestock settings. We use a tractable and scalable methodology to examine the relationship between plasmid metadata and network communities. This reveals how niche (sampling compartment and host genera) partition and shape plasmid diversity. We also perform pangenome-style analyses on network communities. We show that such communities define unique combinations of core genes, with limited overlap. Building plasmid phylogenies based on alignments of these core genes, we demonstrate that plasmid accessory function is closely linked to core gene content. Taken together, our results suggest that stable F-type plasmid backbone structures can persist in environmental settings while allowing dramatic variation in accessory gene content that may be linked to niche adaptation. The association of F-type plasmids with AMR may reflect their suitability for rapid niche adaptation.

## Introduction

Environmental (non-clinical and non-human) populations of *Enterobacterales* may act as a genetic reservoir for antimicrobial resistance (AMR). This includes livestock [1–5] and water-borne [6] resistance. Frequent horizontal gene transfer (HGT) in *Enterobacterales* populations results in a large and open pangenome, enabling the wide-spread transmission of the genes conferring AMR [7–9]. This includes AMR transmission between humans and the environment and vice versa [10]. However, evidence for this transmission is often context and sequence type (ST)-specific, with broader transmission patterns less conclusive [10, 11]. Replicon typing is a plasmid classification system based on well-conserved replication machinery [12]. F-type plasmids are a diverse group of *Enterobacterales*-associated plasmids characterised by their corresponding replicons’ need for DNA gyrase, DnaB, DnaC, DnaG and single-strand binding and DNA polymerase III proteins to replicate [13]. In particular, their involvement in the dissemination of genes encoding extended-spectrum β-lactamases (ESBLs), such as *bla*_CTX-M-15_, is of major clinical concern [14, 15], and almost 40% of plasmid-borne carbapenemases are carried on F-type plasmids [16]. Additionally, F-type plasmids can also carry clinically important virulence genes [17] and colicin genes, sometimes together [18]. F-type plasmids are low copy-number and can be conjugative [19]. Further, recent database analysis suggests F-type replicons are carried in over 50% of multireplicon plasmids [20].

Previous studies of F-type plasmids have often focussed on clinically relevant isolates, often only those encoding ESBLs [16]. Further, they have been limited to studies with smaller sample sizes. Here, we analyse hundreds of F-type plasmids drawn from a survey of environmental diversity in *Enterobacterales*, sampled in 2017 from a region of South-Central England, UK [21]. Sampling was from livestock (cattle, pig and sheep), and from influent, effluent and upstream/downstream waterways surrounding wastewater treatment works (collectively termed WwTWs). Potential seasonal variation was accounted for by sampling over three time-points (TPs) at each site. This provided a high-quality dataset of *n* = 726 plasmids for characterising natural plasmid populations.

Frequent co-integration, recombination and the actions of insertion elements mean the evolution of complete plasmids cannot simply be described with a phylogenetic tree. Instead, networks based on sequence similarity can be used [22]. In such networks, nodes represent plasmids, and edges are weighted by a metric on the plasmid sequences. This captures both vertical and horizontal evolution at the cost of not providing a most recent common ancestor. Communities are a topological property of networks. They are defined as subsets of nodes with dense intra-connections, but sparse inter-connections [23]. In our analyses, they represented groups of similar plasmid sequences. Detecting these structures gives a coarse-grained view of the plasmid population. Previous efforts have often focussed on the relationship between network features used in plasmid classification schemes, such as replicon presence, MOB-type or predicted mobility [24–27]. Further, studies have often focussed on curated selections from online databases [24, 27–29]. It is yet to be seen if similar community structure is present in large-scale, natural populations. In addition, it is important to develop fast and scalable methods for analysis of large and diverse whole genome shotgun datasets. Here we aimed to provide a framework applicable to such studies.

## Results

### A natural population of complete plasmids with F-type replicons

We recovered *n* = 726 circularised plasmids containing an F-type replicon (see Table S1) from a large dataset of high-quality *Enterobacterales* genomes, obtained by hybrid assembly using both short-read (Illumina, 150 bp paired-end) and long-read (PacBio or Nanopore) sequencing of cultured isolates [21]. These isolates were collected over three TPs in 2017 from a region of south-central England, UK. Sampling was from 14 livestock farms (4 pig, 5 cattle and 5 sheep) and from waterways (influent, effluent and rivers) surrounding five WwTWs. Of the livestock plasmids, 120 were from pigs, 137 were from cattle and 150 were from sheep. The remaining 319 plasmids were from WwTWs.

**Fig. 1:**
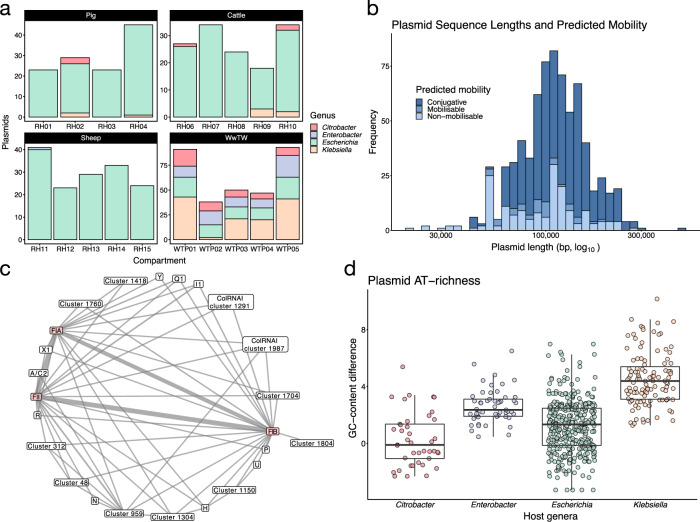
Overview of plasmid population. **a** Plasmid host genera distribution by compartment. **b** Distribution of plasmid sequence lengths with predicted mobilities. **c** Graph representing the association between replicon alleles. F-type nodes are coloured pink. Line weight is proportional to frequency of association in the sample. **d** Plasmid GC-content subtracted from host chromosome GC-content. A value greater than zero indicates the plasmid is AT-richer than the host. Only plasmids with circularised host chromosomes were used (565/726).

F-type plasmids were found across all four of the genera collected in the dataset: *Citrobacter* (53 *C. freundii*), *Enterobacter* (67: 65 *E. cloacae*, 2 untyped *Enterobacter* sp.), *Escherichia* (471 *E. coli*), and *Klebsiella* (135: 61 *K. oxytoca*, 67 *K. pneumoniae* and 7 untyped *Klebsiella* sp.). Livestock plasmids mostly came from *Escherichia* (392/407), whereas WwTW plasmids had a more uniform distribution over all four genera in line with the greater diversity of genera in WwTW isolates (Fig. 1a). Our plasmids originated from *n* = 558 hosts *Enterobacterales* isolates.

Plasmids ranged in length from approximately 20 to 480 kb (Fig. 1b). Most plasmids were predicted to be conjugative (516/726), with a smaller number predicted to be mobilisable (39/726) or non-mobilisable (171/726) (see “Materials and methods”). All plasmids predicted to be conjugative were larger than 42 kbp, consistent with the complete *tra* region of F-type plasmids being approximately 33 kbp [30]. We found 24 different replicons across all plasmids, including 11 in unspecified gene clusters, present in 52 different combinations or ‘replicon haplotypes’ (Table S2). Twenty-two replicon haplotypes appeared only once in the sample. Plasmids carried between 1 and 5 replicons, with a majority carrying 2 (328/726) or 3 (258/726). Plasmid length was positively associated with a number of replicons carried (one-way ANOVA test [*F*(4, 721) = 7.34, *p* value = 8.6e−6] followed by Tukey’s HSD). All plasmids contained at least one F-type replicon (see “Materials and methods”; Fig. S1): FII (574), FIB (460) and FIA (445). Of the remaining replicons, I1 was most common (28), and was always found with an FII replicon. We observed different replicon co-occurrence patterns (Fig. 1c), with individual F-type replicons associated with different non-F-type replicons. For instance, U and N replicons were only found with FIB and FII, respectively. Overall, these co-occurrence patterns corroborate previously observed patterns of frequent F-type association with replicons such as I1, X and R [20].

F-type plasmids tended to be AT-rich relative to their host chromosomes. This trend has been widely reported before [31, 32]. However, we found that relative AT-richness significantly varied between host genus (one-way ANOVA test [*F*(3, 561) = 111, *p* value < 2e−16] followed by Tukey’s HSD), independently of average host GC-content, with *Klebsiella* plasmids having a greater relative AT-richness than other *Enterobacterales* plasmids (Fig. 1d).

### Detecting communities in plasmid *k*-mer networks

**Fig. 2:**
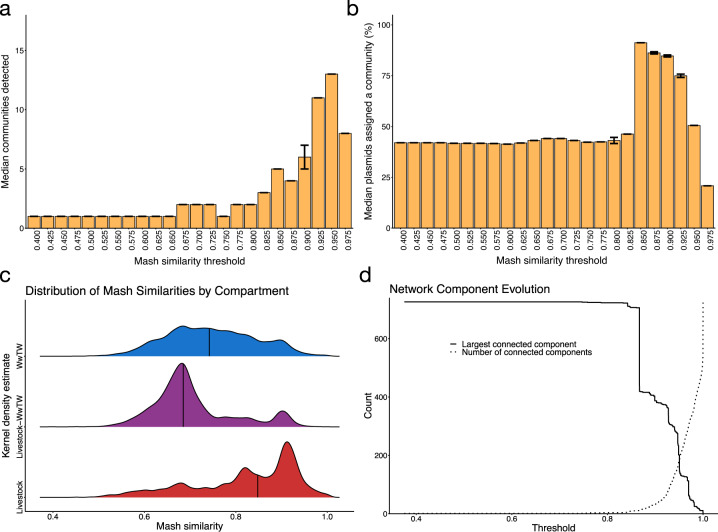
Thresholding the plasmid network. **a** Number of communities (at least 10 nodes) detected over a varying Mash similarity threshold. Median and IQR bar shown. **b** Cumulative proportion of nodes recruited in a detected community of at least ten nodes over a varying Mash similarity threshold. Median and IQR bars shown. **c** Gaussian kernel density estimates of Mash similarities stratified by compartment. Bandwidth = 0.00864 calculated by Silverman’s ‘rule of thumb’. Density medians are indicated with vertical lines. **d** Evolution of the largest connected component and number of components over a varying Mash similarity threshold.

Plasmid sequence distances were calculated using Mash, a *k*-mer based distance estimation [33] (ranges from 0 to 1, 0 being approximately identical; see “Materials and methods”). We used the similarities (1—Mash distance) as weighted edges in a plasmid network. The output Mash edge list is presented in Table S3. Communities were detected using the Louvain algorithm, which optimises the modularity of the networks, and is a weighted community detection algorithm, meaning it also accounts for the Mash similarities [23]. The all versus all comparison of sequences produced a network too dense for consistent performance from each Louvain run (Fig. 2). Hence, we reduced the density of our network by thresholding the edges (i.e. by ‘sparsification’). This involves removing all edges below a fixed Mash threshold. The necessity of sparsification in plasmid networks has been noted before [25, 27]. We considered several statistics to optimise our network threshold: (i) the number of communities detected (Fig. 2a), (ii) the proportion of plasmids recruited into communities (Fig. 2b) and (iii) kernel density estimates (KDEs) of network edge weights stratified by sampling compartment (Fig. 2c). To ensure the communities represented potential sub-populations, we only considered those with at least ten plasmids. Figure S2 shows statistics (i) and (ii) for communities with at least three plasmids. The large drop in community recruitment seen at threshold = 0.825 (Fig. 2b) was due to the break-up of a large connected component (Fig. 2d). Note the statistics in Fig. 2a, b are averaged over 100 runs of Louvain to account for the algorithm converging to different local optima along the boundaries of overlapping communities. The Louvain algorithm first assigns a different starting community to each node [23] i.e. different random seeds produce different starting configurations. Because the first step is a greedy algorithm which first locally optimises modularity, different starting communities can lead to different final communities, particularly at community boundaries, so averaging overruns is a common technique when using the Louvain algorithm. This variation is reflected in the IQR bars in Fig. 2a, b.

**Fig. 3:**
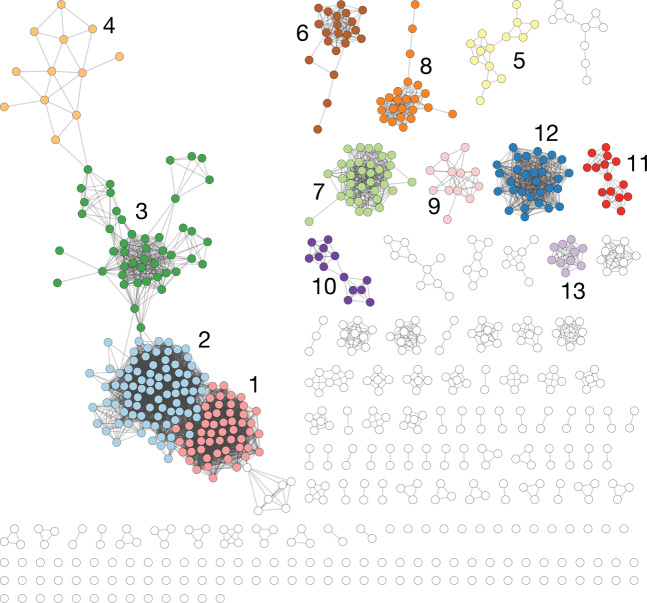
Plasmid network communities. The plasmid network at threshold = 0.95. Each community with at least ten members has a unique colour. Communities are labelled from 1 to 13, which correspond to Figs. 5, S3–4 and S5–15. Unassigned plasmids and those in smaller communities are left white.

We selected a threshold = 0.95, which yielded the highest number of communities (13) containing at least 10 plasmids (Fig. 2a), and coverage of over 50% (Fig. 2b). Figure 2c highlighted that livestock plasmid (median = 0.85) were generally more similar to each other than WwTW plasmids (median = 0.74) and suggested that plasmid diversity was higher in WwTW isolates. At our threshold = 0.95, we revealed the structure of livestock plasmids at the expense of minimal WwTW structure break-up. At this level, the network’s largest connected component (LCC) had 201 nodes with 182 connected components in total (Fig. 2d). There were 99 singleton plasmids, consistent with high levels of diversity in the population. A visualisation of the network at this threshold with the 13 communities coloured is presented in Fig. 3. The quality of communities was validated using the normalised mutual information score (NMI; see “Materials and methods”) against MOB-cluster IDs (NMI = 0.73) and replicon haplotypes (NMI = 0.55). Closer inspection revealed that most communities were dominated by a single or multiple closely related replicon haplotypes and MOB-cluster IDs (Figs. S3–4; community members and validation metadata is given in Table S4). This suggests that our methodology accurately assigns plasmid communities.

### Community metadata analysis

To evaluate the relationship between the node metadata labels and the network, two entropic measures were considered: homogeneity (*h*) and completeness (*c*) (both range from 0 to 1; see “Materials and methods”). Homogeneity measures the distribution of labels given a community, with an ideal community containing a single label: a high homogeneity means that plasmids with similar sequences tend to have similar metadata labels. Conversely, completeness measures the distribution of communities given a label: high completeness means that instances of a label tend to fall within a single community. Importantly, both homogeneity and completeness are independent of community size, the number of communities, and the number of metadata labels. This makes the approach robust to uneven sampling strategies, such as the disproportionate number of *E. coli* isolates in our sample.

Each plasmid was assigned a set of metadata labels, consisting of a sampling compartment (livestock type [pig, cattle, sheep] or WwTW-association [influent, effluent, upstream and downstream]), a host genus (*Citrobacter*, *Enterobacter*, *Escherichia* or *Klebsiella*), and a TP (1, 2 or 3). Homogeneity (Table 1) and completeness (Table 2) were averaged over 100 runs of the Louvain algorithm. Despite the number of communities remaining consistent, some variation in the measures arose from minor changes in community boundaries.

**Table 1:** Community metadata homogeneity.

	Mean ± sd homogeneity						
Median ± IQR communities with at least 10 plasmids	Livestock, WwTW	Pig, Cattle, Sheep, WwTW	14 Livestock Farms, WwTW	Livestock, 5 WwTWs	Livestock, Upstream/Influent, Downstream/Effluent	Host Genera	Time-point
13 ± 0	0.713 ± 0.014	0.592 ± 0.006	0.406 ± 0.000	0.468 ± 0.032	0.553 ± 0.009	0.888 ± 0.000	0.050 ± 0.000

Homogeneity score averages over 100 runs of the Louvain algorithm for all 13 communities.

**Table 2:** Community metadata completeness.

	Mean ± sd completeness						
Median ± IQR communities with at least 10 plasmids	Livestock, WwTW	Pig, Cattle, Sheep, WwTW	14 Livestock Farms, WwTW	Livestock, 5 WwTWs	Livestock, upstream/influent, downstream/effluent	Host genera	Time-point
13 ± 0	0.200 ± 0.001	0.332 ± 0.000	0.400 ± 0.000	0.238 ± 0.002	0.211 ± 0.003	0.309 ± 0.000	0.023 ± 0.000

Completeness score averages over 100 runs of the Louvain algorithm for all 13 communities.

**Fig. 4:**
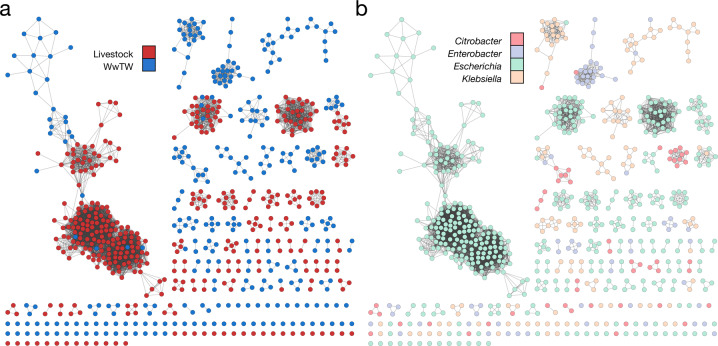
Plasmid network coloured by metadata. All nodes are coloured, not just those in our detected 13 communities of at least 10 members. **a** Partition by livestock or WwTW sampling compartment. **b** Partition by plasmid host genera.

Homogeneity scores showed that the sampling compartment shaped plasmid similarity. At the coarsest resolution, there was high homogeneity considering livestock versus WwTW (*h* = 0.713; Table 1), meaning that plasmid communities were largely distinct between livestock and WwTW settings. This metadata partition is projected on the network in Fig. 4a. However, homogeneity was lower when comparing different livestock types (pig, cattle and sheep) (*h* = 0.592) and even more so when comparing different farms (*h* = 0.406), meaning that there was a loss of structure at these levels and plasmid communities were not well segregated by the individual farm. Homogeneity was also low if plasmids were stratified by individual WwTWs (*h* = 0.468). However, homogeneity increased for influent/upstream versus effluent/downstream compartments (*h* = 0.553) indicating some differences in plasmids before and after WwTW treatment. Overall, plasmids from WwTWs were weakly structured by wastewater catchment.

Completeness scores highlighted higher WwTW diversity compared to lower livestock diversity. For the binary livestock or WwTW label plasmid communities scored low completeness (Table 2; *c* = 0.200), which changed little when stratified over the individual WwTWs (*c* = 0.238), indicating a uniform distribution of WwTW labels over the plasmid communities and high diversity. Based on our Mash similarity KDEs (Fig. 2c), we would expect livestock plasmids to have higher completeness scores than WwTW plasmids due to the lower levels of diversity; as anticipated, when stratifying the livestock metadata, completeness scores increased (*c* = 0.332 and *c* = 0.400). This indicated plasmids from the same farm were more likely to be found in the same community.

Host genus also played an important factor in partitioning plasmid diversity. The homogeneity scores were very high, implying a significant genetic partition by the host (Table 1; *h* = 0.888). This metadata partition is displayed in Fig. 4b. The lower completeness suggested a moderate level of diversity across all *Enterobacterales* plasmids (Table 2; *c* = 0.309). There was a very weak TP effect found in the network (Tables 1 and 2; *h* = 0.050 and *c* = 0.023). Under a one-tailed permutation test, all metadata label configurations except TP had a zero *p* value for homogeneity and completeness (Table S5; see “Materials and methods”), indicating that overall, there was a significant association between niche (sampling compartment and host genus) and plasmid population structure.

### Community pangenomes

To explore the genetic structure of the communities we considered the set of all represented genes within a community, known as the pangenome. Plasmids had a median of 35 annotated genes (range: 4–112). Genes conferring AMR were found in 17% (122/726) of plasmids; this included 33 plasmids carrying ESBLs (9 pig, 8 cattle and 16 WwTw), with 4 carrying *bla*_CTX-M-15_ (all WwTW). F-type plasmids in isolates cultured from pigs were disproportionately associated with AMR genes (45/109 [41%] AMR plasmids).

Core genes with well-conserved synteny comprise the plasmid ‘backbone’ [22], which often controls essential replication and mobility functions. Genes with accessory function, such as AMR genes, are inserted into the backbone. For 13 F-type plasmid communities identified in this study using the 0.95 thresholds above (see Fig. 3), we found a median of 13 core genes (range: 0–88; Table 3). Each community possessed a unique combination of core genes, and pairs of communities shared a median of 0 core genes between them (range: 0–21) (Table S6). The communities had a median of 463 accessory genes (range: 151–790), sharing a median of 284 accessory genes (range: 99–570) (Table S7). Pairs of communities sharing a higher number of genes tended to have a higher sum of individual genes (*r* = 0.81, *t* = 12.95, *p* value < 2.2e−16), indicating an overlap between larger pangenomes. Within a plasmid community, we found a greater mean Mash similarity was associated with more core genes (*r* = 0.63, *t* = 2.70, *p* value = 0.02) and a lower total number of genes in the pangenome (*r* = −0.67, *t* = −3.00, *p* value = 0.01).

**Table 3:** Community pangenomes.

Community	Nodes	Edges	Mash similarity mean	Core genes	Soft core genes	Shell genes	Cloud genes	Total genes
1	52	1151	0.973	13	12	155	153	333
2	85	1935	0.968	4	17	140	383	544
3	46	325	0.965	35	8	86	369	498
4	12	21	0.962	2	0	290	129	421
5	14	23	0.962	2	0	225	260	487
6	21	111	0.963	13	6	354	430	803
7	34	263	0.966	2	1	278	359	640
8	23	135	0.978	27	1	142	362	532
9	12	34	0.966	18	0	364	324	706
10	13	37	0.977	0	0	309	38	347
11	15	55	0.981	62	0	116	35	213
12	30	391	0.976	68	3	126	187	384
13	12	45	0.978	88	0	195	48	331

Characteristics of each of the 13 communities, including a number of nodes, edges and Mash mean (mean weight of all edges), and gene counts at each level of the pangenome: core genes, softcore genes, shell genes and cloud genes are those found in [100, 99], (99, 95], (95, 15], and (15, 0] per cent of plasmids, respectively.

**Fig. 5:**
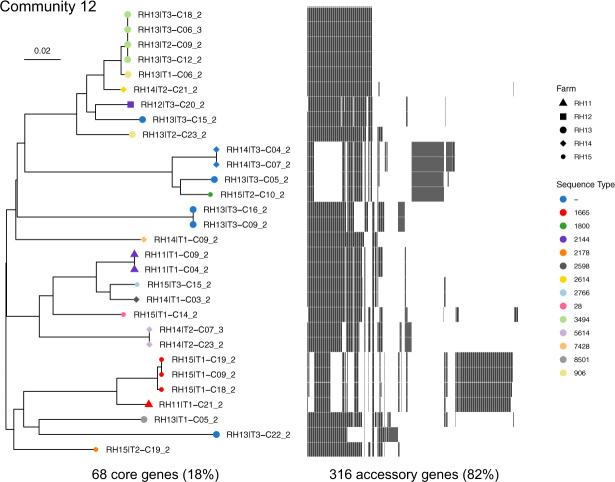
Community core gene phylogeny. A neighbour-joining tree based on alignments of the 68 core genes. A heatmap of the 316 accessory genes is also shown. Node colour represents a host sequence type and node shape represents the farm. Unknown STs are labelled by ‘-’. Branch lengths have been corrected for homologous recombination.

For an example community of 30 F-type plasmids from isolates from sheep farms, we produced a neighbour-joining phylogeny based on 64/384 core genes (Fig. 5). The tree accounts for homologous recombination, with events detected in 11/30 leaf nodes and 21 internal nodes, consistent with a high number of exchange events affecting this plasmid community. The median tract length was 156 bp (range: 2–2249 bp). Annotation of the phylogeny with the 316 accessory genes for this community revealed that accessory gene presence aligned almost identically with the core gene phylogeny, suggesting that the evolution of the plasmid backbone is highly linked to accessory function. All host genera for this plasmid community were diverse *E. coli*, with 13 known STs present, consistent with the widespread horizontal transfer of the plasmids from this community. Within this community, no plasmids carried AMR genes. Core genome phylogenies for other plasmid communities also showed a strong link between accessory gene presence and backbone contents (Figs. S5–S15).

## Discussion

We have analysed plasmid communities using alignment-free genomic networks to explore diversity within a large, natural population of F-type plasmids from four *Enterobacterales* genera (*Citrobacter*, *Enterobacter*, *Escherichia* and *Klebsiella*). These F-type plasmids contained a diversity of replicons (plasmids contained 21 other replicons, forming 62 unique combinations) and we resolved plasmids into communities (13 communities of ≥10 plasmids). We found that 15% of F-type plasmids contained at least one AMR gene, and 5% carried an ESBL. This underlines that non-clinical plasmid populations can also carry AMR genes and that WwTW environment and livestock niches are part of an AMR network for *Enterobacterales* [2, 10].

Our network analysis revealed F-type plasmids were well partitioned by sampling compartment, with distinct communities isolated to WwTWs or livestock; however, there were also clear instances of sharing events between, for example, specific farm locations. There was also moderate partitioning by specific livestock species: pig, cattle and sheep. In addition, there was a difference in plasmids before and after WwTW treatment. Sampling compartment also influenced diversity, with a higher diversity in WwTW-associated plasmids than livestock plasmids. This is probably because both river and wastewater catchments integrate a large number of human, livestock (farmed and wild) and environmental sources. Despite F-type plasmids ranging over all *Enterobacterales* genera, it suggested some genus-specific adaptations. Notably, the extent of plasmid-host AT-richness relative to the host chromosome varied depending on the genus. It remains to be seen how such observed differences relate to plasmid function. However, this may be related to the livestock–WwTW partition, since our livestock plasmids were predominantly hosted by *E. coli*. We did not detect an effect of sampling TP. This is maybe because our TPs were too close and our sample size too small to capture any significant evolution, or it may indicate that time of year is not a strong factor in determining community structure. It would be interesting to see how plasmids from clinical samples relate to those from our samples within the network, especially if pre-WwTW plasmids are considered as a proxy for human gut microbiomes.

Pangenome analysis of the inferred plasmid communities revealed that core gene content was mostly unique to communities. Further, they were strongly related to accessory function. Taken with the above results, we propose that the sampling compartment and host greatly influence the function of plasmids. This includes AMR presence, with pigs, and hence *Escherichia*, carrying a disproportionate burden in our sample. The pangenomes for communities varied greatly in the number of core genes, with one community having zero. This may be because the similarity threshold was not severe enough to resolve this particular community into multiple similar groups, or also may have resulted from the settings used in Panaroo (see “Materials and methods”) which may have split homologous gene clusters. Generally, more genetically similar communities had a greater number of core genes and smaller pangenome. Our results for F-type plasmid communities are in line with a recent study of the wider prokaryotic plasmidome which concluded that clusters of plasmids contain common genomic backbones [29].

Our study has several limitations. One important limitation, which applies more widely to network approaches which cluster or partition diversity, is that thresholding of the network is somewhat arbitrary and highly dataset dependent. Trade-offs are required to reveal the intermediate structures of the network whilst maintaining good community detection performance. We determined a threshold by considering Mash similarity distributions and component evolution alongside Louvain output diagnostics but were focused on recovering communities of more than ten plasmids. For a different purpose e.g. investigating HGT between communities, the full network could be studied. When diversity varies greatly between sampling compartments, a single threshold is unlikely to be globally optimal. In these cases, it is probably best to focus on subpopulations of interest. Despite only considering several hundred nodes here, our methodology is scalable to far larger studies. Originally, the Louvain algorithm had runtime complexity *O*(*e*), where *e* is the number of edges in the network. This has since been improved to *O*(*v* log *k*), where *v* is the number of nodes and *k* is the average node degree [34]. Further, recent efforts have parallelised the Louvain algorithm to networks with billions of edges, though this approach was not necessary here [35]. Although Acman et al. [27] argued that Louvain was unsuitable for the large and dense plasmid networks they investigated, we believe it may be appropriate for future analyses. Finally, our dataset is limited to the four *Enterobacterales* genera understudy and conclusions may not reflect the wider diversity of F-type plasmids beyond these genera.

Our study adds to the growing literature on genomic plasmid networks to characterise and partition diversity. To our knowledge, ours is the first study to analyse the network structure of a large-scale (*n* = 726), natural plasmid population, and to focus specifically on F-type plasmids. Whereas previous studies have based plasmid networks on sequence alignments [24], or the sharing of annotated genes [25] and open reading frames [29], we adopted an approach similar to Acman et al. [27] and Jesus at al. [28] using alignment-free Mash distances. These prior studies analysed all publicly available plasmid sequences deposited in the NCBI’s RefSeq database and are therefore likely subject to any biases associated with sequence deposition in this catalogue. This is in contrast to the dataset studied here, where we characterised a large number of plasmids and their relationships within a clearly defined, local sampling frame. While previous studies used other algorithms such as OSLOM [27] and stochastic block modelling [29] for community detection, we have demonstrated the Louvain algorithm as a viable alternative for plasmid networks.

In conclusion, our approach used a high-resolution strategy for summarising similarities and differences within plasmid populations, using the advantages of having complete plasmid sequences and analysing these in the context of associated metadata. For F-type plasmids, we were able to show the distinct, local effects of sampling compartment on plasmid structure and population. We were also able to identify evidence for sharing of plasmids between bacterial lineages, farms and WwTW-associated contexts, with relevance for the ‘One Health’-associated study of mobile genetic elements and AMR genes. As long-read sequencing costs fall, and increasingly large numbers of plasmids can be characterised, future work applying this method will contribute to better understanding plasmid populations, estimating transfer rates of important AMR genes and MGEs between potential reservoirs, and identifying hotspots of selection/transfer that might be amenable to intervention.

## Materials and methods

Plasmids and corresponding host isolates were sampled and sequenced on behalf of the REHAB project in 2017, which aimed to characterise the non-clinical, non-human *Enterobacterales* microbiome in south-central England, with a focus on better understanding AMR spread. Specifically, livestock (pig farms, cattle farms and sheep farms) and WwTWs (influent, effluent, upstream and downstream waterways) were sampled. To account for seasonal variation, sampling occurred at three discrete TPs: January–April 2017 (TP1), June–July 2017 (TP2) and October–November 2017 (TP3). All the plasmids presented have at least one F-type replicon (classified by with MOB-typer, see below). In total, we present *n* = 726 plasmids originated from *n* = 558 isolates. This comprises a subset of the entire REHAB dataset, which overall contains *n* = 2293 circularised plasmids recovered from *n* = 828 isolates. This dataset is described in more detail [21].

### Livestock

Four pig farms (RH01–04), five cattle (RH06–10) and five sheep farms (RH11–15) were selected for sampling over all three TPs. All participating farmers provided written consent for participation. Specific details on farm recruitment and sampling procedure can be found in [21] and Anjum et al. (paper in preparation).

### WwTWs environment

Five WwTWs (WTP01–05) were selected based on a number of criteria, including geographic location within the region, wastewater treatment configuration, wastewater population equivalent served, consented flow, and the accessibility of the effluent receiving river for sampling both upstream and downstream. The chosen WwTWs and their details are shown in Table S8. Sampling took place over all three TPs. Specific details are provided in [21].

### DNA sequencing

The isolates were selected for sequencing to represent diversity within the four major genera (*Citrobacter*, *Enterobacter*, *Escherichia* and *Klebsiella*) in each niche, including the use of third-generation cephalosporin resistance to identify a subset of multi-drug resistant isolates within each genus. Sequencing involved either PacBio SMRT (*n* = 125 chromosomes; *n* = 163 plasmids) or Oxford Nanopore Technologies (ONT) (*n* = 433 chromosomes; *n* = 563 plasmids) methodologies. Specific details are provided in ref. [21].

### Genome assembly, assignment and typing

We used the hybrid assembly and sequencing methods described in our pilot study [36] to produce high-quality *Enterobacterales* genomes from short and long reads. We assigned species and ST from assembled genomes using mlst (version 2.16.43) [37]. Further details on validation are provided in [21].

### Plasmid assembly

We used the hybrid assembly and sequencing methods described in a pilot study [36] to produce high-quality *Enterobacterales* genomes with associated plasmids from short and long reads. The Illumina short reads helped resolve the smaller plasmids, which were not very repetitive. In short, we used Unicycler (version 0.4.7) [38] with ‘normal’ mode, --min_component_size 500, --min_dead_end_size 500, and otherwise default parameters. From these, we selected *n* = 726 plasmids which contained an F-type replicon after classification with MOB-typer (see below). We searched all plasmids against PLSDB (version 2020-03-04) [39] which contains 20,668 complete published plasmids, using Mash screen [40] and keeping the top hit. All plasmids had a match.

### Replicon and predicted mobility typing

We used MOB-typer from MOB-suite (version 2.0.0) [26]. We clustered plasmids using MOB-cluster IDs and assigned replicon types with MOB-typer, both part of the MOB-suite. MOB-cluster uses single linkage clustering with a cutoff of a Mash distance of 0.05 (corresponding to 95% ANI). MOB-typer predicts mobility based on of annotations of relaxase (*mob*), mating pair formation (MPF) complex, and *oriT* genes [26] In short, a plasmid is putatively labelled conjugative if it has both relaxase and MPF, mobilisable if it has either relaxase or *oriT* but no MPF, and non-mobilisable if it has no relaxase and *oriT*. A recent large-scale study [20] showed MOB-typer to have a higher correct classification rate than the widely used PlasmidFinder [41]. Figure S1 provides a neighbour-joining phylogeny of all F-type replicon sequences used by MOB-typer. We used replicon sequence Mash distances [33] with a *k*-mer length of 13 and a sketch size of 5000, followed by ggtree (version 3.11) [42] to visualise the phylogeny. Replicon sequences AY04580 | IncFIC, CP003035|IncFIC, 000136__AP014877_00014|IncFIA and 000097_NC_025116|IncFIB had branch lengths rescaled to zero due to a negative branch length artefact from the neighbour-joining tree algorithm. This may be due to the high diversity between the replicon sequences. Alternative replicon typings are provided by PlasmidFinder [41] (Table S9; using Abricate version 1.01 [43] with PlasmidFinder database version 2020-07-13) and PlasmidMLST [44] on PubMLST (Table S10; version 1).

### Plasmid similarity estimation

Distances between the complete plasmid sequences were calculated using Mash (version 2.2) [33]. We then used 1—Mash distances to obtain the similarities. Mash reduces sequences to a fixed-length MinHash sketch, which is used to estimate the Jaccard index. This measures extent of *k*-mer sharing between plasmids. The representative sketch is far shorter than the original sequence, making distance calculations efficient over large datasets. It also gives the Mash distance (range = 0,1 with 0 being ~identical sequences and 1 being ~completely dissimilar sequences). Mash assigns each pair-wise sequence distance a *p* value of that distance (or less) under the null hypothesis both sequences are random. A *k*-mer length of 13 and a sketch size of 5000 was used. All other settings were default. Using Mash considerably reduces similarity computation time from exact *k*-mer profile methods, whilst maintaining good performance. The Mash output is provided in Table S3.

### Louvain community detection

The Louvain algorithm detects communities by optimising the modularity by iterative expectation–maximisation [23]. This aims to maximise the density of edges within communities against edges between communities. The algorithm was implemented using the python-Louvain (version 0.14) Python module.

### Community validation

NMI (range = 0,1 with 1 being a perfect match) measures the information that the community labels and either MOB-cluster IDs or replicon haplotypes share [45]. NMI was calculated using the R package ‘aricode’ [46]. Community labels used are same as those used to produce the community phylogenies (see Table S4).

### Community metadata analysis

Homogeneity (*h*) and completeness (*c*) are dual conditional entropy-based measures [47]. They are independent of the clustering algorithm, dataset size, number of label-types, number of communities and community sizes. This means they are appropriate for uneven metadata distributions. A community partition satisfies homogeneity (*h* = 1) if all members have the same metadata label-type. Suppose we have a network with *N* nodes, partitioned by a set of metadata labels, $M = \{ m_i|i = 1, \ldots ,n\}$, and a set of communities, $C = \{ c_j|j = 1, \ldots ,m\}$. Let $A = \{ a_{ij}\}$ represent the *ij-*th entry in the contingency table of partitions. Hence, *a*_*ij*_ counts the number of nodes with label *m*_*i*_ in community *c*_*j*_. We then say

$$h = \left\{ {\begin{array}{*{20}{c}} 1 \\ {1 - \frac{{H\left( {M\left| C \right.} \right)}}{{H\left( M \right)}}} \end{array}} \right.\begin{array}{*{20}{c}} {{\mathrm{if}}\;H(M,C) = 0} \\ {{\mathrm{else}}} \end{array}$$

where

$$H\left( {M\left| C \right.} \right) = - \mathop {\sum}\limits_{c = 1}^{\left| C \right|} {\mathop {\sum}\limits_{m = 1}^{\left| M \right|} {\frac{{a_{mc}}}{N}\log \frac{{a_{mc}}}{{{\sum} {_{c = 1}^M} a_{mc}}}} }$$

and

$$H\left( M \right) = - \mathop {\sum}\limits_{m = 1}^{\left| M \right|} {\frac{{{\sum} {_{c = 1}^{\left| C \right|}} a_{mc}}}{n}\log \frac{{{\sum} {_{c = 1}^{\left| C \right|}} a_{mc}}}{n}}$$

are the conditional entropy of the metadata given the communities and the entropy of the communities, respectively $H(M|C) = 0$ when the community partition coincides with the metadata partition, and no new information is added. A community partition satisfies completeness (*c* = 1) if all instances of a metadata label-type are assigned the same community. Completeness is defined dually by

$$c = \left\{ {\begin{array}{*{20}{c}} 1 \\ {1 - \frac{{H\left( {C\left| M \right.} \right)}}{{H\left( C \right)}}} \end{array}} \right.\begin{array}{*{20}{c}} {{\mathrm{if}}\;H(C,M) = 0} \\ {{\mathrm{else}}} \end{array}$$

The measures were calculated using the scikit-learn (version 0.22.2) Python module [48].

### Permutation test

We first calculated a Louvain partition for the network and selected all nodes in communities with at least 10 members. Homogeneity and completeness score medians were used from Table 1 and Table 2. The partition labels were then randomly permuted 1000 times. For each permutation, the homogeneity and completeness scores were calculated. These were then used to calculate a right-tailed *p* value. The results are shown in Table S5.

### Plasmid annotation and pangenome analysis

Plasmids were annotated using Prokka (version 1.14.6) [49]. Pangenome analysis used Panaroo (version 1.2.2) [50]. Core genes, softcore genes, shell genes and cloud genes are those found in [100, 99], (99, 95], (95, 15], and (15, 0] per cent of sequences respectively. Within the pangenome, core genes are typically defined as those shared by ≥99% of constituent plasmids. However, since no plasmid community in this study had >100 members, core genes were strictly shared by 100%. Under 50 Louvain trials, only one partition was different, where RH11|T2-C24_4 was assigned community 1 instead of 2. This is to be expected since communities 1 and 2 overlaps (Fig. 3). The community labels for the pangenome analysis are from when this does not happen (see Table S4). AMR annotations used Abricate (version 0.9.8) [43] with the NCBI AMRFinder Plus database [51] with a threshold of 90% sequence identity and 90% coverage. AMR annotations are provided in Table S11.

### Community phylogeny

Alignment of core genes used Clustal Omega (version 1.2.4) [52], and ClonalFrameML (version 1.2) [53] was used to adjust for homologous recombination. We used ggtree (version 3.11) [42] to visualise the phylogeny.

### Data visualisation

All figures were made in using the R package ggplot2 (version 3.3.0) [54], except for the network Figs. (1c, 3 and 4a, b), which were made using Cytoscape (version 3.8.0) [55]. Cytoscape was also used to calculate some network descriptive statistics.

## Supplementary information

Supplementary Materials

Table S1

Table S3

Table S4

Table S9

Table S10

Table S11

## Data Availability

Plasmid sequence data, metadata (Table S1), Mash edge list (Table S3), community validation metadata (Table S4), PlasmidFinder output (Table S9), Plasmid MLST output (Table S10) and Abricate NCBI output (Table S11) are available in a figshare collection (10.6084/m9.figshare.c.5066684.v3). Other data can be found in ref. [21].

## Code availability

Details on computing methods can be found in the GitHub repository for the paper (https://github.com/wtmatlock/plasmid-network-analysis). This includes scripts for calculating the LCC and NCCs, Louvain performance diagnostics and running the permutation test.
