# Supplementary material for: Genomic network analysis of environmental and livestock F-type plasmid populations
Source: ISME J. 2021 Mar 1;15(8):2322–35. doi: 10.1038/s41396-021-00926-w (PMC8319146; doi:10.1038/s41396-021-00926-w)
Supplement: Supplementary file 1 — Supplementary Materials [file 41396_2021_926_MOESM1_ESM.docx]

**Genomic network analysis of environmental and livestock F-type plasmid populations**

Supplementary materials

**Supplementary figures:**

Figure S1: Phylogeny for MOB-typer F-type replicon sequences

Figure S2: Louvain performance for communities with at least 3 members.

Figure S3: Community composition by MOB-typer cluster IDs

Figure S4: Community composition by replicon haplotype

Figures S5-S-15: Plasmid core-gene phylogenies for communities 1, 2, 3, 4, 5, 6, 7, 8, 9, 11, and 13

**Supplementary tables:**

Table S2: ‘Replicon haplotype’ counts for the *n*=726 IncF plasmids

Table S5: ­*p*-values for the permutation test on homogeneity and completeness scores

Table S6: Matrix counting the number of shared core genes between communities 1-13

Table S7: Matrix counting the number of shared accessory (non-core) genes between communities 1-13

Table S8: Metadata for the 5 WwTW sampling locations

**Figure S1. Neighbour joining phylogeny of MOB-typer database F-type replicons.** Replicon sequences AY04580|IncFIC, CP003035|IncFIC, 000136__AP014877_00014|IncFIA and 000097_NC_025116|IncFIB had branch lengths rescaled to zero due to a negative branch length artefact from the neighbour joining tree algorithm.

a

b

**Figure S2. Louvain performance for communities with at least 3 members.** (a) Number of communities detected over a varying Mash similarity threshold. (b) Percentage of plasmids recruited into a community over a varying Mash similarity threshold.

**Figure S3. Community composition by MOB-typer cluster IDs.**

**Figure S4. Community composition by replicon haplotype.**

**Figure S4. Neighbour joining phylogeny of MOB-typer database F-type replicons.** Replicon sequences AY04580|IncFIC, CP003035|IncFIC, 000136__AP014877_00014|IncFIA and 000097_NC_025116|IncFIB had branch lengths rescaled to zero due to a negative branch length artefact from the neighbour joining tree algorithm.

Community 1

**Figure S5. Plasmid core-gene phylogeny for community 1.**

Community 2

**Figure S6. Plasmid core-gene phylogeny for community 2.**


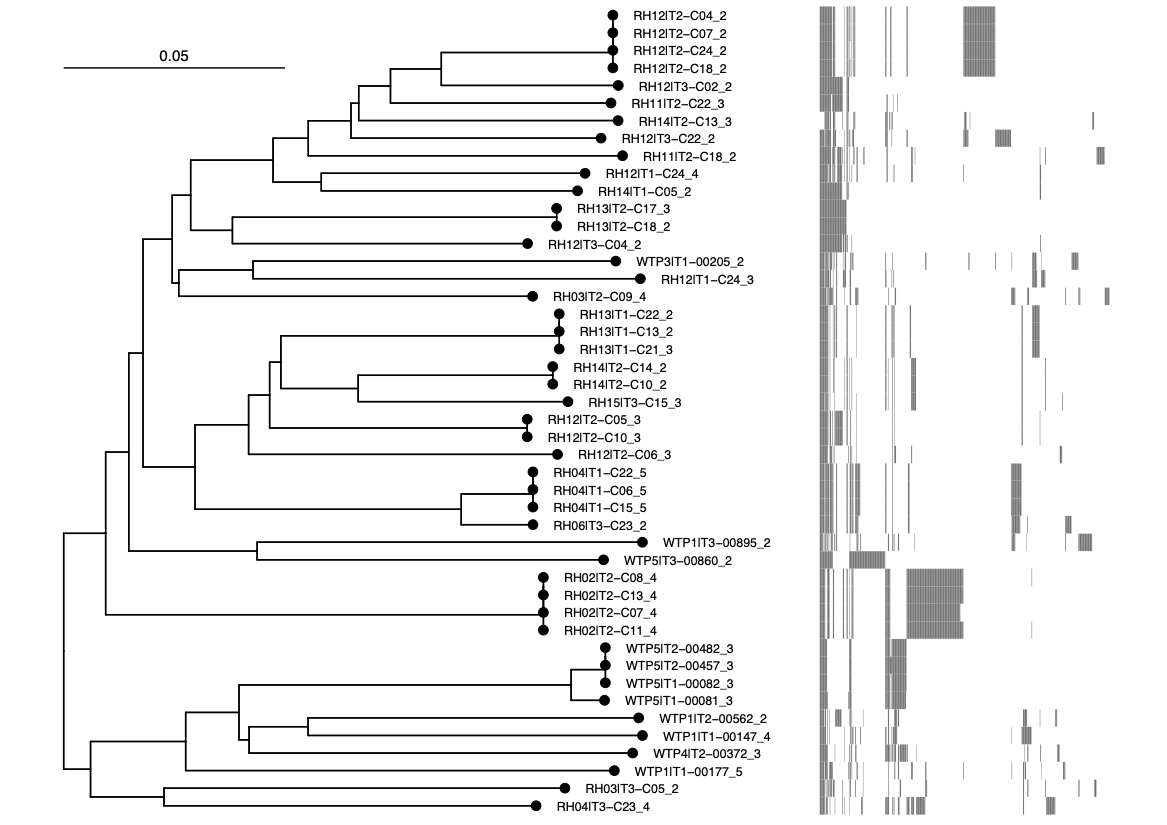


Community 3

**Figure S7. Plasmid core-gene phylogeny for community 3.**


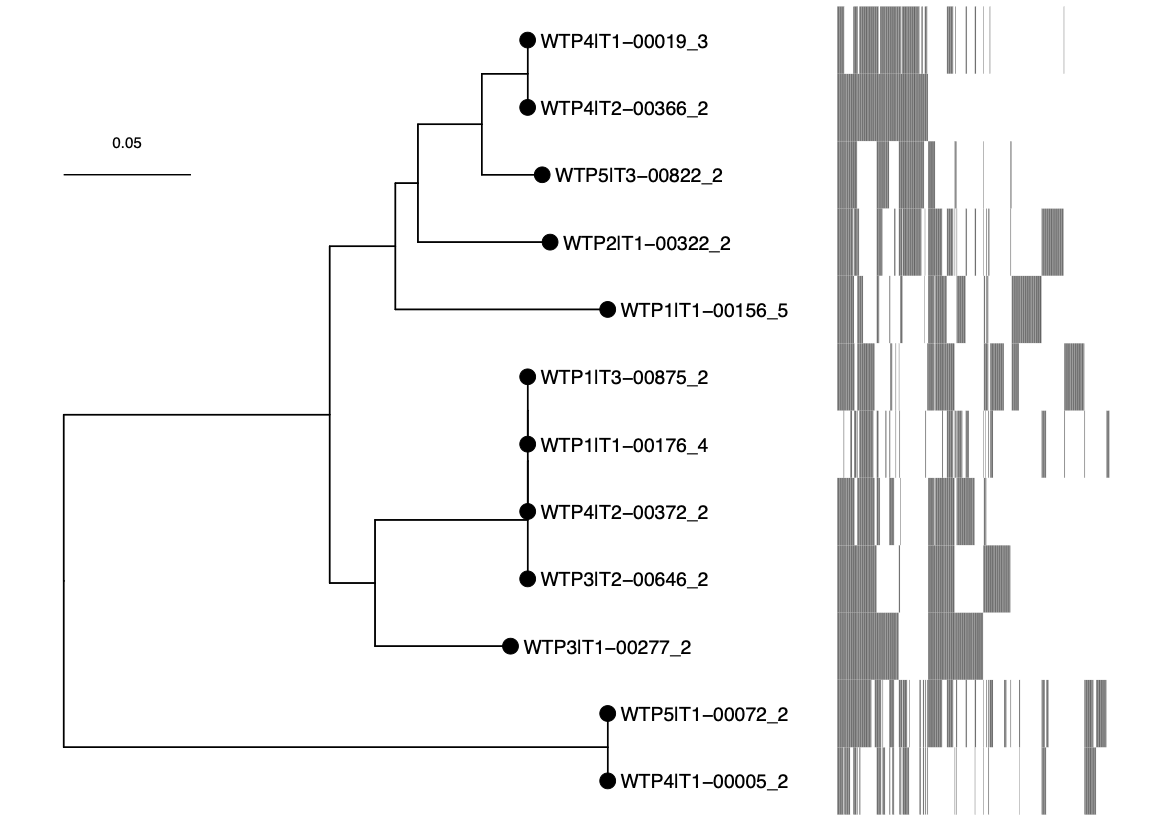


Community 4

**Figure S8. Plasmid core-gene phylogeny for community 4.**

Community 5


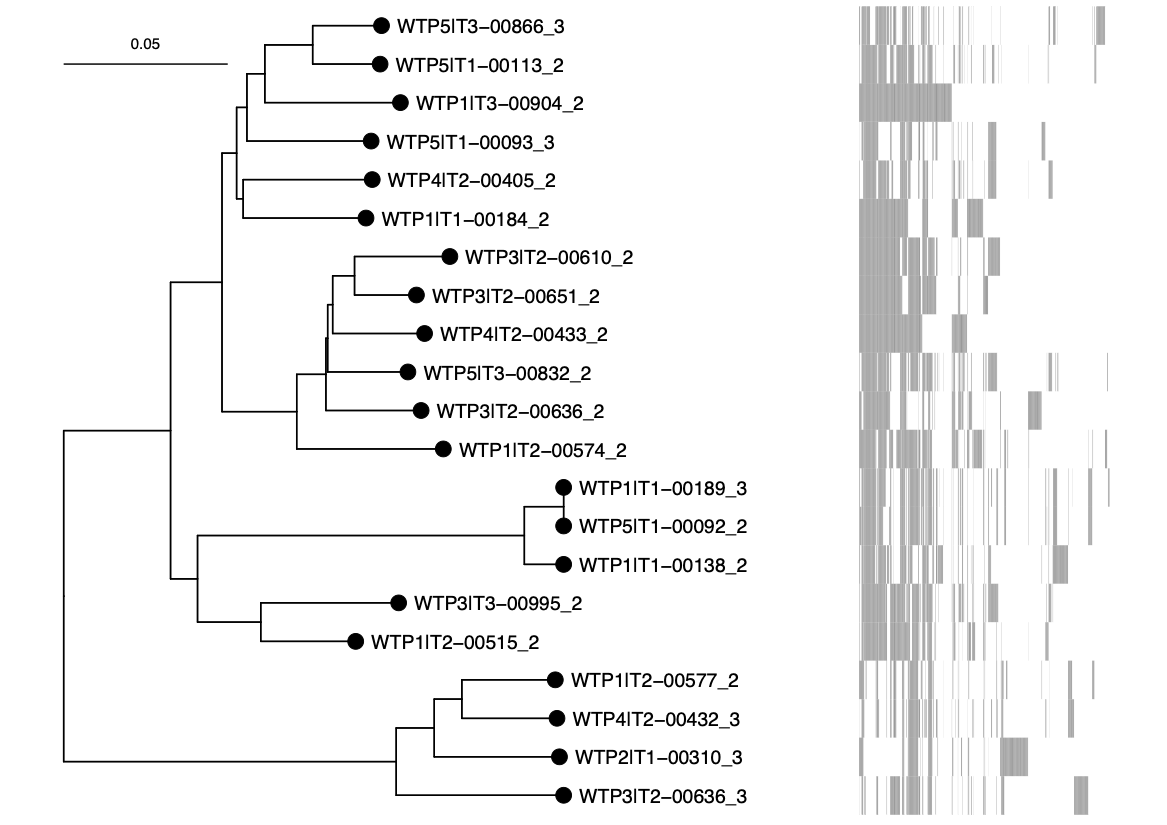


**Figure S9. Plasmid core-gene phylogeny for community 5.**

Community 6


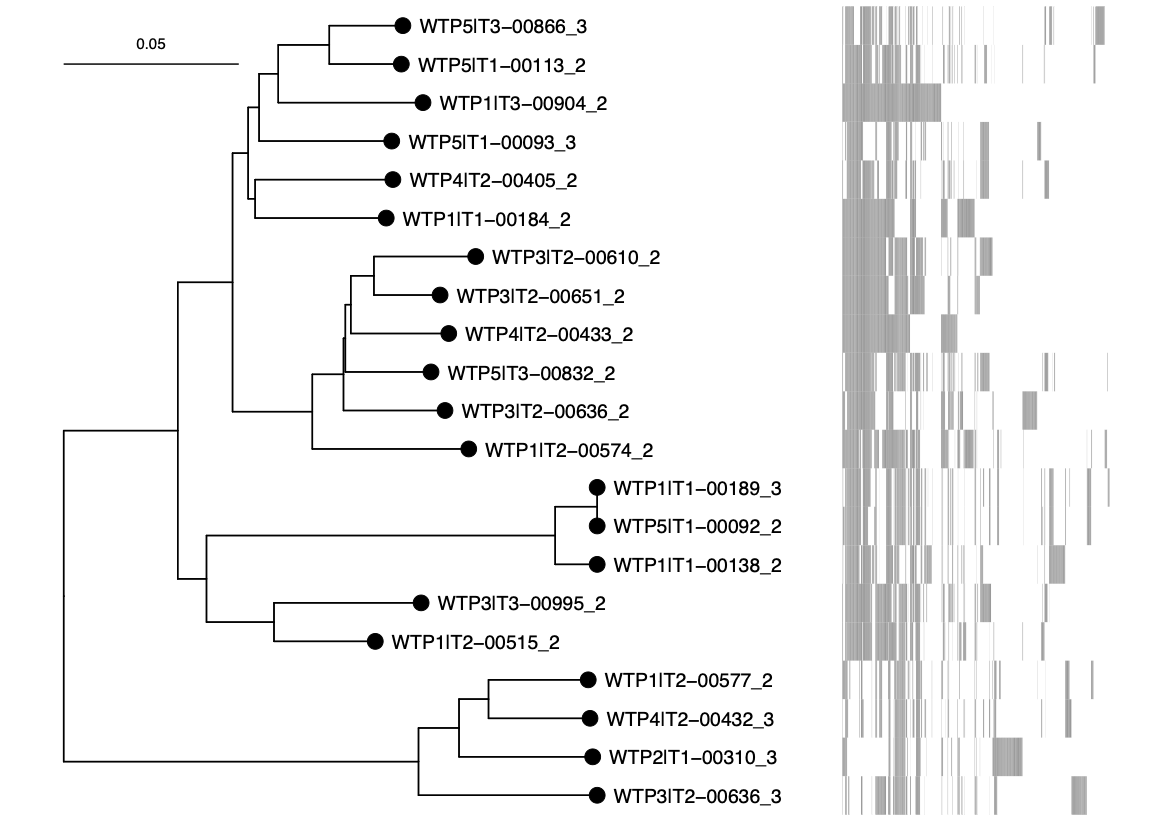


**Figure S10. Plasmid core-gene phylogeny for community 6.**

Community 7


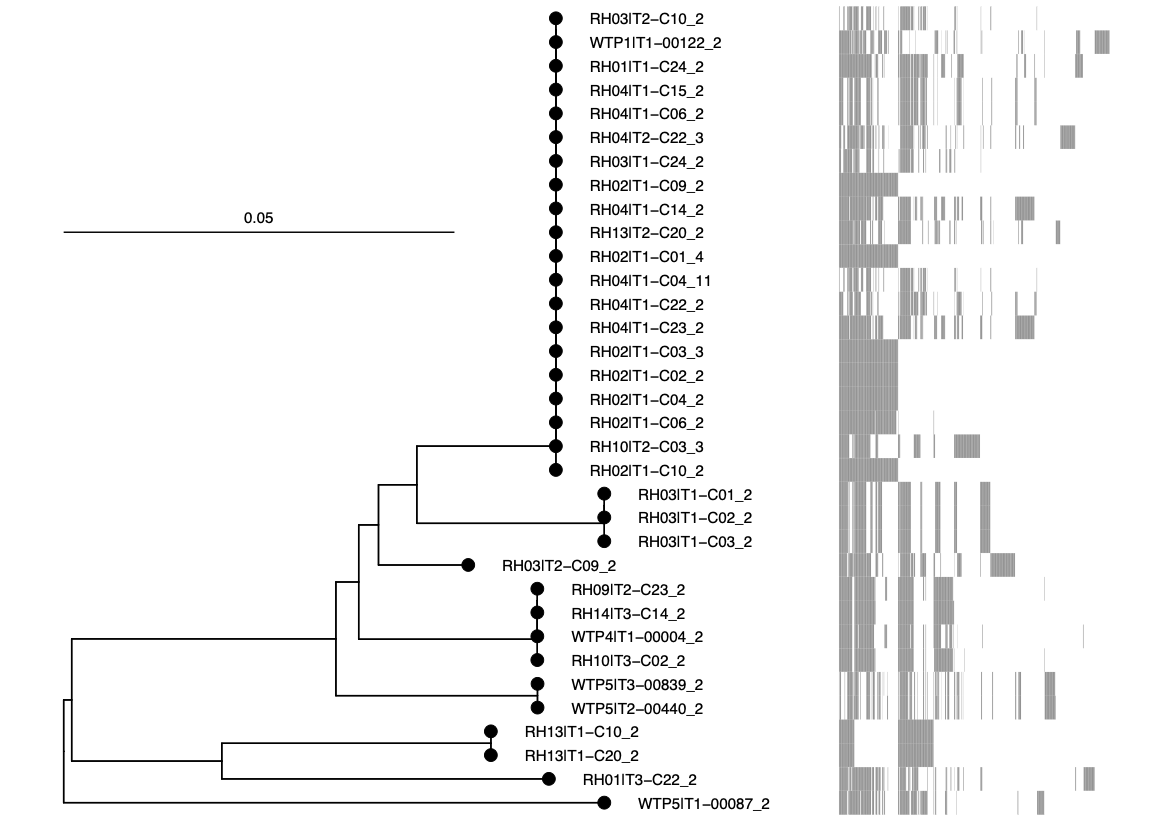


**Figure S11. Plasmid core-gene phylogeny for community 7.**


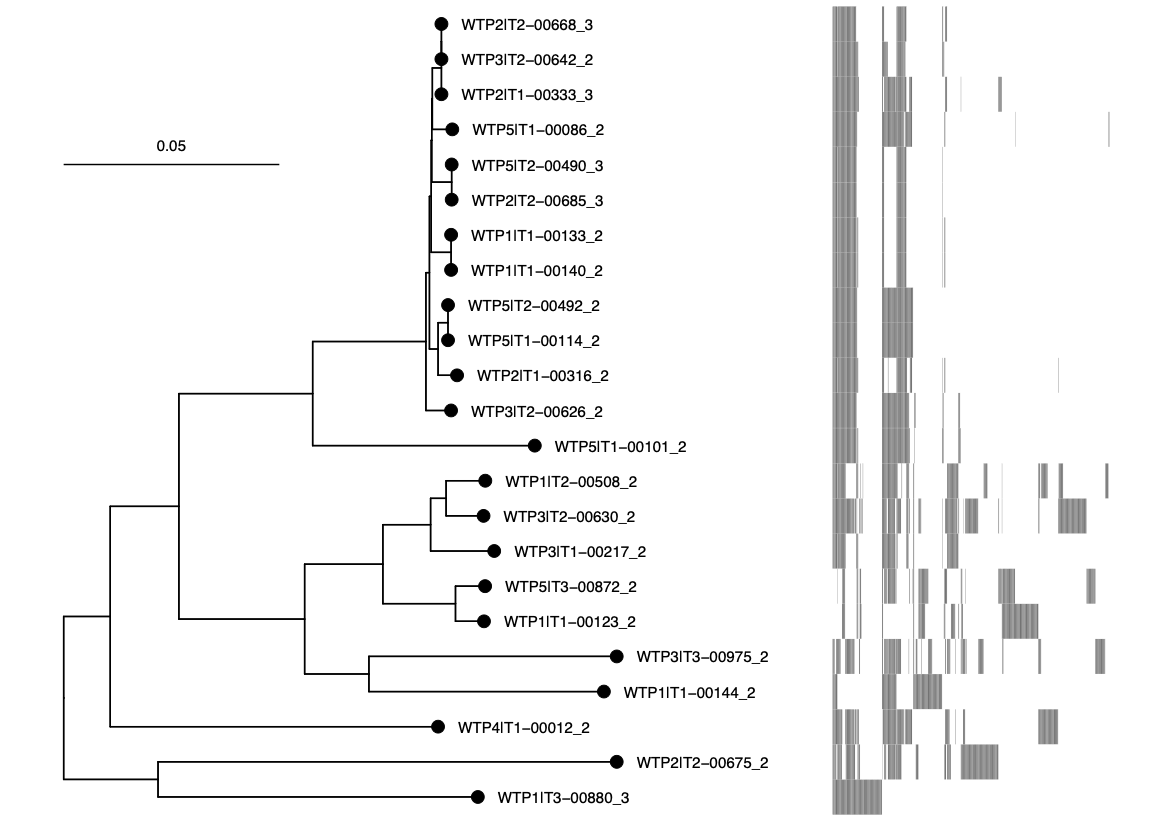


Community 8

**Figure S12. Plasmid core-gene phylogeny for community 8.**


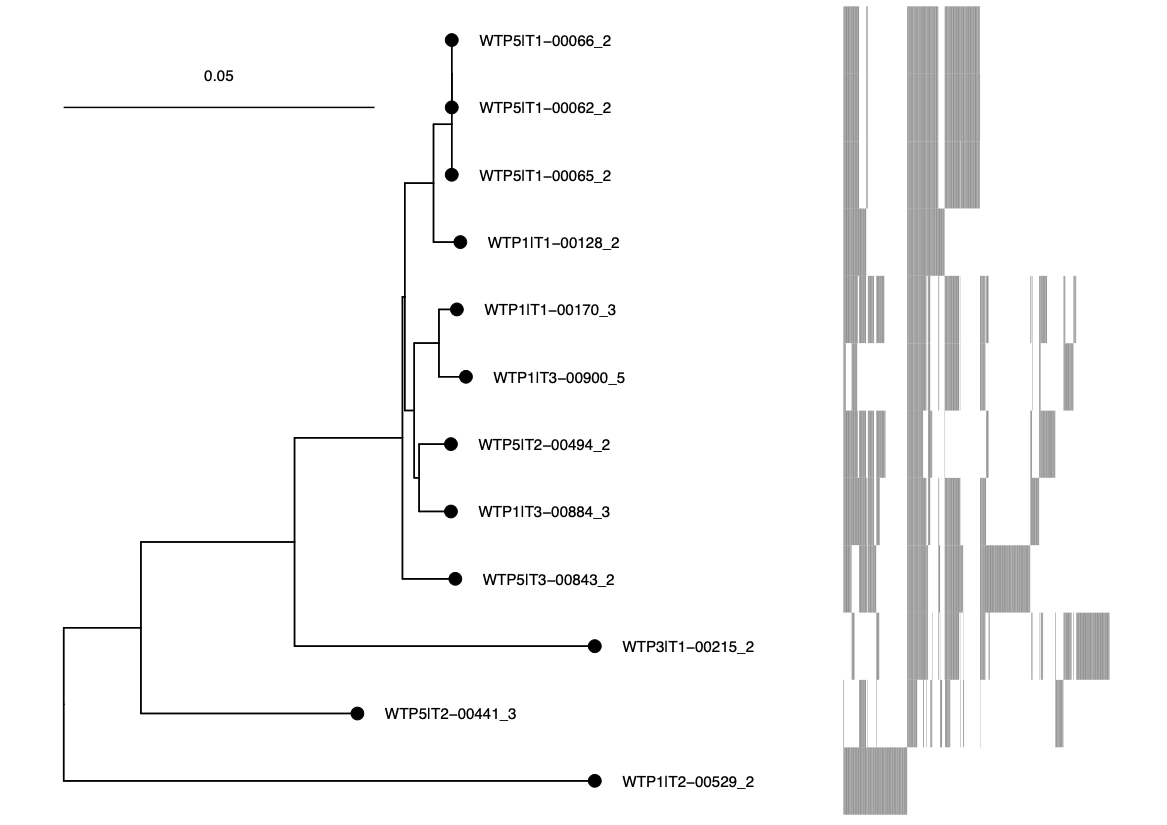


Community 9

**Figure S13. Plasmid core-gene phylogeny for community 9.**


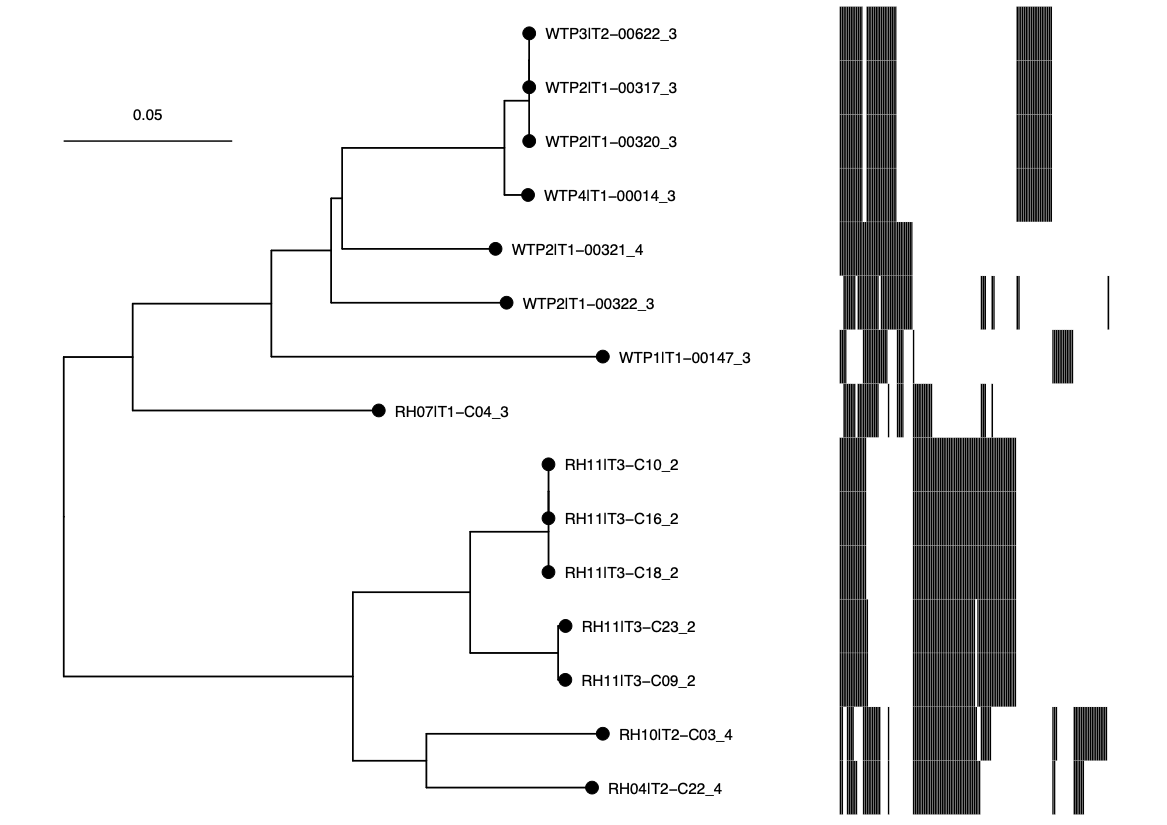


Community 11

**Figure S14. Plasmid core-gene phylogeny for community 11.**


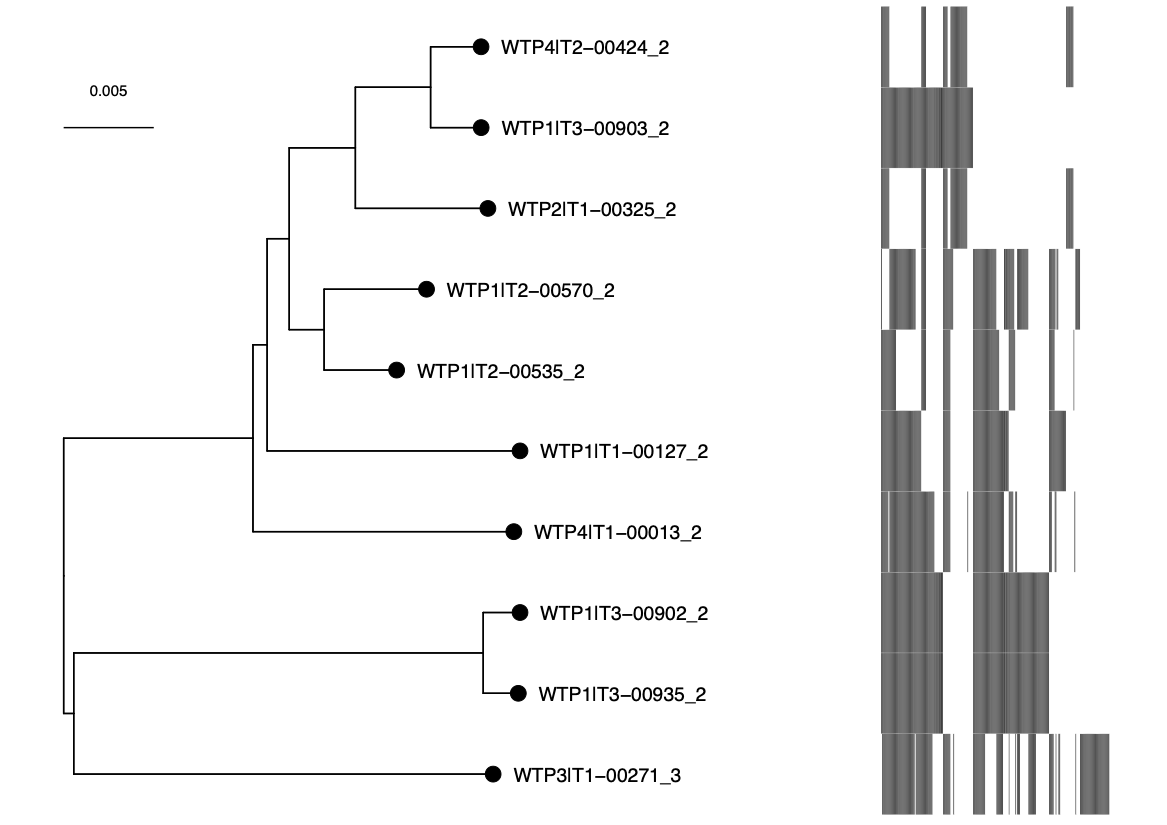


Community 13

**Figure S15. Plasmid core-gene phylogeny for community 13.**

| Table S2: Replicon Haplotypes | | | |
| --- | --- | --- | --- |
| Haplotype | **Count** | Haplotype | Count |
| FIA, FIB, FII | 214 | FIA, Y | 2 |
| FIA, FII | 126 | FIB, FII, rep_cluster_48 | 2 |
| FIB, FII | 91 | FIB, FII, rep_cluster_959 | 2 |
| FIB | 65 | FII, rep_cluster_312, rep_cluster_48, rep_cluster_959 | 2 |
| FII | 30 | ColRNAI_rep_cluster_1987, FIA | 1 |
| FIA, FIB | 26 | ColRNAI_rep_cluster_1987, FII, rep_cluster_959 | 1 |
| FII, rep_cluster_959 | 23 | A/C2, FII, rep_cluster_959 | 1 |
| FIA | 20 | FIA, FIB, FII, H, rep_cluster_1304 | 1 |
| FII, I1 | 16 | FIA, FIB, FII, X1 | 1 |
| FIA, Q1 | 10 | FIA, FIB, FII, rep_cluster_1304 | 1 |
| FIA, FIB, rep_cluster_1760 | 9 | FIA, FIB, FII, rep_cluster_1704 | 1 |
| FII, rep_cluster_1418 | 9 | FIA, R | 1 |
| ColRNAI_rep_cluster_1291, FIA, FIB, FII | 8 | FIA, rep_cluster_959 | 1 |
| FIB, FII, I1 | 7 | FIB, FII, P | 1 |
| FIB, rep_cluster_1150 | 7 | FIB, FII, R | 1 |
| FII, R | 6 | FIB, FII, rep_cluster_312, rep_cluster_48 | 1 |
| FIA, FIB, FII, Q1 | 5 | FIB, H, rep_cluster_1150 | 1 |
| FIA, rep_cluster_1418 | 4 | FIB, P | 1 |
| ColRNAI_rep_cluster_1987, FIB, FII | 3 | FIB, R | 1 |
| FIA, FIB, FII, I1 | 3 | FIB, U, rep_cluster_1150 | 1 |
| FIA, FII, rep_cluster_959 | 3 | FIB, Y | 1 |
| FIB, FII, rep_cluster_312 | 3 | FIB, rep_cluster_1804 | 1 |
| FIA, FIB, FII, R | 2 | FII, N, rep_cluster_959 | 1 |
| FIA, FII, I1 | 2 | FII, rep_cluster_1304, rep_cluster_959 | 1 |
| FIA, FII, R | 2 | FII, rep_cluster_312 | 1 |
| FIA, FII, X1 | 2 | FII, rep_cluster_48, rep_cluster_959 | 1 |

| Table S5: Permutation Test | | |
| --- | --- | --- |
| Metadata Labels | Homogeneity *p*-value | Completeness *p*-value |
| Livestock, WwTP | *p*<0.0001 | *p*<0.0001 |
| Pig, Cattle, Sheep, WwTP | *p*<0.0001 | *p*<0.0001 |
| 14 Livestock Farms, WwTP | *p*<0.0001 | *p*<0.0001 |
| Livestock, 5 WwTPs | *p*<0.0001 | *p*<0.0001 |
| Livestock, Influent/Upstream, Effluent/Downstream | *p*<0.0001 | *p*<0.0001 |
| Host Genera | *p*<0.0001 | *p*<0.0001 |
| Time-point | 0.033 | 0.035 |

| Table S6: Community Core Gene Set Intersections | | | | | | | | | | | | | | |
| --- | --- | --- | --- | --- | --- | --- | --- | --- | --- | --- | --- | --- | --- | --- |
|  |  | **2** | **3** | **4** | **5** | **6** | **7** | **8** | **9** | **10** | **11** | **12** | **13** |  |
| **1** | 13 |  |  |  |  |  |  |  |  |  |  |  |  |  |
| **2** | 0 | 4 |  |  |  |  |  |  |  |  |  |  |  |  |
| **3** | 0 | 1 | 35 |  |  |  |  |  |  |  |  |  |  |  |
| **4** | 0 | 1 | 0 | 2 |  |  |  |  |  |  |  |  |  |  |
| **5** | 0 | 0 | 0 | 0 | 2 |  |  |  |  |  |  |  |  |  |
| **6** | 1 | 0 | 0 | 0 | 0 | 13 |  |  |  |  |  |  |  |  |
| **7** | 0 | 1 | 0 | 2 | 0 | 0 | 2 |  |  |  |  |  |  |  |
| **8** | 0 | 0 | 2 | 0 | 0 | 0 | 0 | 27 |  |  |  |  |  |  |
| **9** | 0 | 0 | 0 | 0 | 0 | 0 | 0 | 13 | 18 |  |  |  |  |  |
| **10** | 0 | 0 | 0 | 0 | 0 | 0 | 0 | 0 | 0 | 0 |  |  |  |  |
| **11** | 2 | 0 | 2 | 0 | 0 | 1 | 0 | 0 | 0 | 0 | 62 |  |  |  |
| **12** | 3 | 1 | 10 | 0 | 0 | 2 | 0 | 0 | 0 | 0 | 7 | 68 |  |  |
| **13** | 2 | 1 | 7 | 1 | 2 | 2 | 1 | 1 | 0 | 0 | 21 | 15 | 88 |  |

| Table S7: Community Accessory Gene Set Intersections | | | | | | | | | | | | | | |
| --- | --- | --- | --- | --- | --- | --- | --- | --- | --- | --- | --- | --- | --- | --- |
|  | 1 | 2 | 3 | 4 | 5 | 6 | 7 | 8 | 9 | 10 | 11 | 12 | 13 |  |
| 1 | 320 |  |  |  |  |  |  |  |  |  |  |  |  |  |
| 2 | 295 | 540 |  |  |  |  |  |  |  |  |  |  |  |  |
| 3 | 281 | 397 | 463 |  |  |  |  |  |  |  |  |  |  |  |
| 4 | 285 | 344 | 343 | 419 |  |  |  |  |  |  |  |  |  |  |
| 5 | 278 | 382 | 355 | 345 | 485 |  |  |  |  |  |  |  |  |  |
| 6 | 275 | 454 | 372 | 345 | 418 | 790 |  |  |  |  |  |  |  |  |
| 7 | 285 | 474 | 409 | 366 | 393 | 509 | 638 |  |  |  |  |  |  |  |
| 8 | 267 | 400 | 366 | 334 | 385 | 429 | 405 | 505 |  |  |  |  |  |  |
| 9 | 278 | 455 | 379 | 350 | 405 | 570 | 519 | 424 | 688 |  |  |  |  |  |
| 10 | 273 | 304 | 300 | 313 | 314 | 310 | 311 | 302 | 314 | 346 |  |  |  |  |
| 11 | 126 | 131 | 132 | 142 | 134 | 132 | 142 | 131 | 138 | 128 | 151 |  |  |  |
| 12 | 247 | 283 | 263 | 263 | 255 | 255 | 263 | 250 | 254 | 246 | 123 | 316 |  |  |
| 13 | 177 | 185 | 181 | 188 | 212 | 203 | 190 | 190 | 199 | 186 | 99 | 167 | 243 |  |

| Table S8 | | | | | |
| --- | --- | --- | --- | --- | --- |
| WwTW | Population equivalient (PE) | Primary treatment | Secondary treatmant | Tertiary treatment | Consented Flow (m^3^/d) |
| WTP01 | 223,435 | PSTs | ASP | N/A | 50,985 |
| WTP02 | 49,522 | PSTs | ASP | Disc filters | 11,883 |
| WTP03 | 37,731 | PSTs | ASP | Sand filters | 11,476 |
| WTP04 | 26,905 | PSTs | Filters | N/A | 6,250 |
| WTP05 | 2,841 | PSTs | Filters | N/A | 2,000 |
